# Agricultural and Physiological Responses of Tomato Plants Grown in Different Soilless Culture Systems with Saline Water under Greenhouse Conditions

**DOI:** 10.1038/s41598-019-42805-7

**Published:** 2019-05-01

**Authors:** Wilbert M. Rodríguez-Ortega, Vicente Martínez, Manuel Nieves, I. Simón, V. Lidón, J. C. Fernandez-Zapata, J. J. Martinez-Nicolas, José M. Cámara-Zapata, Francisco García-Sánchez

**Affiliations:** 1 Centro de Edafología y Biología Aplicada del Segura, Consejo Superior de Investigaciones Científicas, Campus Universitario de Espinardo, 3A, 30100 Murcia, Spain; 2 Escuela Politécnica Superior de Orihuela, Universidad Miguel Hernández, Carretera de Beniel, km 3.2, 03312 Alicante, Spain

**Keywords:** Plant physiology, Abiotic

## Abstract

Tomato is the most important horticultural crop in the world. The yields for this crop are highest in Southeastern Spain. In this work we studied a commercial variety of tomato, with different soilless culture systems (deep flow technique, nutrient film technique, and the perlite substrate) and three levels of salinity (2.2, 6.3, and 10.2 dS·m^−1^) typical of Southeastern Spain. The irrigation management was carried out for optimizing the water use efficiency. Alterations in the water status of the plants, Cl^−^ and Na^+^ toxicity, and nutritional imbalances altered the vegetative growth and physiology of the plants. The marketable yield was affected by both soilless culture system and salinity. Regarding the soilles culture system, yield decreased in the order: deep flow technique > perlite > nutrient film technique. The salinity treatments improved the fruits quality by increasing the total soluble solids and titratable acidity. Plants cultivated with the nutrient film technique had the highest concentrations of Cl^−^ and Na^+^ and the highest Na^+^/K^+^ ratio. The concentrations of Cl^−^ and Na^+^ in the plants were not related directly to the yield loss. Therefore, the influence of the toxicity, osmotic effect, and nutritional imbalance seems to have been responsible for the yield loss.

## Introduction

On a worldwide basis, tomato is the most important horticultural crop, with a total production and cultivated area estimated at 164 Mt and 4.76 Mha, respectively. China is the main producer, with 31.0% of the total world production and 20.6% of the total cultivated area^1^, while Spain is the country with the highest mean tomato yield (81.3 t·ha^−1^). In Spain, as in other countries, tomato crops are grown mainly in areas having an arid or semi-arid Mediterranean climate, characterized by an average annual rainfall of less than 300 mm. Since tomato is a plant that requires a large amount of water for its development^2^ in areas with a shortage of good-quality water for irrigation farmers use poor-quality water - from wells or groundwater aquifers - containing a high concentration of soluble salts, mainly NaCl, which decreases the agronomic yield.

Soilless production of tomato crops in greenhouses has increased dramatically in recent years. Thus, in Southeastern Spain there are approximately 5,500 ha of soilless culture^3^. This is due to the fact that these systems allow nutrition and irrigation to be controlled more efficiently, which generates higher yields^4–6^. In addition, they reduce the incidence of pathological problems considerably, implying lower initial investment costs, and avoid contamination of soils and aquifers by nitrate and pesticides, thus contributing to the practice of sustainable agriculture^7^. In countries with environmental legislation, the use of these systems has been encouraged in the last 10 years, to minimize the damage to natural ecosystems caused by excessive use of fertilizer^8^.

Tomato plants are considered moderately sensitive to salinity^2^. The growth and yield of the crop begin to decline when the electrical conductivity (EC) of the nutrient solution (NS) with which it is cultivated exceeds 2.5–4.0 dS·m^−1^ ^9^. This level of salinity improves the quality of the fruits, since their concentrations of total soluble solids and antioxidant compounds increase, but reduces vegetative development as well as the water use efficiency and yield^10–15^. The first effect of salinity on plants is the “osmotic effect”, since the roots are exposed to excess salt in the growth medium - which limits the uptake of water, creating a water deficit in the plant that has negative effects on growth. As the time of exposure to salt increases, plants begin to suffer phytotoxicity, due to Cl^−^ and Na^+^ accumulation, and nutritional imbalances, as the uptake of some nutrients is inhibited^11–15^. These three factors will negatively influence the physiological and metabolic processes of plants - such as photosynthesis, respiration, and cell division^16^ - and will lead to the synthesis of reactive oxygen species, which ultimately cause a decrease in vegetative growth and yield^17^.

In soilless culture systems, the irrigation must be sufficient to maintain high evapotranspiration rates and an adequate supply of nutrients, while ensuring adequate levels of oxygen in the root system and low salt accumulation^18,19^. A wide variety of substrates are available commercially, both organic (coconut fiber, wood chips, cereal straws, cork waste, etc.) and inorganic (rock wool, fiberglass, perlite, vermiculite, expanded clay). Floating-root systems, such as hydroponics or the nutrient film technique (NFT), are also used.

Among the soilless culture systems most used in Southeastern Spain for tomato cultivation, the use of perlite, watered with NS but without recirculation, is of particular note. However, the lack of water forces farmers to optimize irrigation management, to maximize production with the lowest water consumption. In this area a new type of NFT system, called the New Growing System (NGS ©), is being commercialized, which reduces the water consumption due to the recirculation of the NS. This system is frequently used in crops such as strawberry or lettuce^20^. Recently, the manufacturer of this new system has optimized its management to produce tomatoes with a saving of water, obtaining a mature and competitive technology. However, the behavior of tomato in different soilless culture systems has not been compared in the conditions typical of the Southeast of Spain, where irrigation with saline water predominates. Therefore, the objective of this work is to compare the agronomic and physiological response of tomato plants in a traditional soilless culture system (perlite) and in this new NFT system, using saline water with EC values similar to those used in the southeast of Spain. In addition, a non-commercial hydroponic crop (deep flow technique, DFT) has been used as a control system that allows us to know the maximum potential of water use efficiency (WUE_agri_, kg·m^−3^) for this crop.

## Results

### Fruit yield and quality

**Figure 1:**
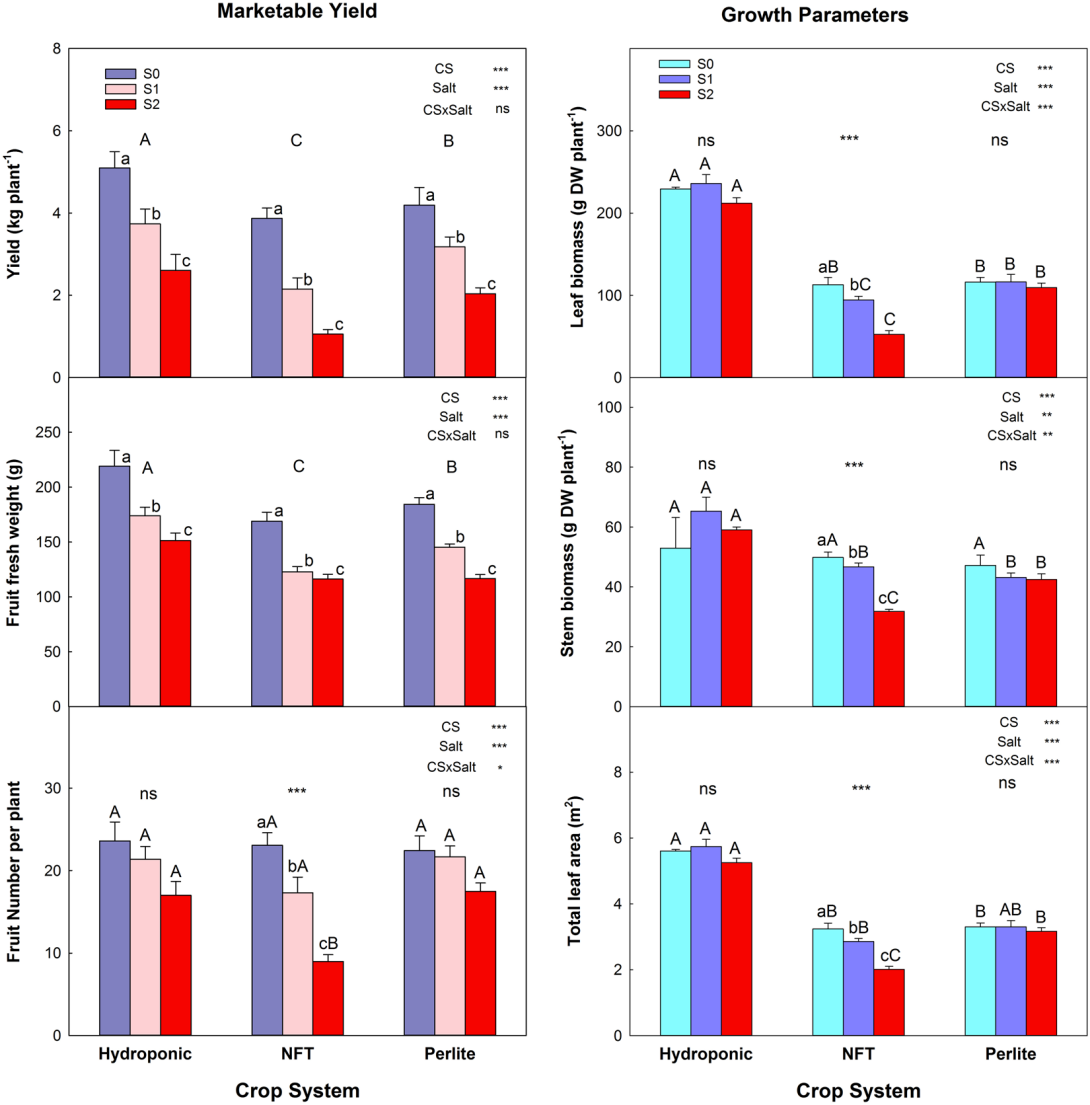
Parameters of marketable yield and vegetative development of tomato plants grown in three soilless culture systems and three salt treatments. The error bar indicates the standard error of the mean (n = 4–8). *, **, ***, and “ns” indicate significant differences at p < 0.05, p < 0.01, p < 0.001, and non-significant differences, respectively. Values with different letters differ significantly at the 95% level, according to Tukey’s HSD test. Lowercase letters compare salinity treatments for each culture system. Uppercase letters compare culture systems for each salinity treatment.

The marketable yield (kg·plant^−1^) was estimated from the total yield, by excluding the fruits having a fresh weight of less than 70 g and those showing symptoms of apical rot, cracking, or any type of deformation or mechanical damage (Fig. 1A).

The marketable yield was affected by both the soilless culture system and the salinity treatment (NS with EC at 2.2, 6.3 and 10.2 dS m^−1^ for treatments S0, S1, and S2, respectively) although no interaction between these two factors was observed. The most productive system, with an average of 5.0 kg·plant^−1^, was the DFT, followed by perlite (4.2 kg·plant^−1^) and NFT (3.8 kg·plant^−1^). With regard to salinity, increasing the NaCl concentration in the NS decreased the yield progressively (Fig. 1A). Tomato plants grown with DFT had higher yields than those of NFT, for each salinity treatment, due to a decrease in the mean fruit weight with treatment S1 and a decrease in the number of fruits with treatment S2 for the NFT plants.

The physical parameters of fruit quality - longitudinal and equatorial diameter, and firmness - varied with both the cultivation system and the salinity treatment; however, there was no interaction between these two factors (Table 1). The fruits from the DFT and perlite systems had higher values of longitudinal and equatorial diameter, and lower values of the shape index, than those of the NFT system. The firmness was greatest in the fruits from the DFT system, followed by perlite and, finally, NFT. The increase in salinity decreased both diameters, equatorial and longitudinal, as well as firmness.

**Table 1:** Fruit quality: equatorial and longitudinal diameters, shape index, hardness, and firmness. For each column, values not sharing a common letter differ significantly at the 95% level, according to Tukey’s HSD test. *, ***, and “ns” indicate significant differences at p < 0.05, p < 0.001, and non-significant differences, respectively, among salinity treatments for each soilless culture system. In all cases, the mean value ± the standard error is shown (n = 4–8).

	Equatorial diameter (mm)	Longitudinal diameter (mm)	Shape index (−)	Hardness (N·m^−2^)	Firmness (N)
**Main factor: System**					
DFT	78.6 ± 2.6a	60.8 ± 1.7a	0.77 ± 0.01b	5.28 ± 0.32	16.03 ± 0.56a
NFT	65.5 ± 2.0b	55.5 ± 1.4b	0.85 ± 0.01a	5.31 ± 0.20	12.15 ± 0.60c
Perlite	76.2 ± 1.9a	60.6 ± 1.4a	0.80 ± 0.00ab	5.08 ± 0.15	14.21 ± 0.60b
**Main factor: Salinity**					
S0	82.6 ± 1.8a	65.2 ± 0.9a	0.79 ± 0.01	5.11 ± 0.21	16.82 ± 0.47a
S1	70.9 ± 1.8b	59.0 ± 1.1b	0.83 ± 0.01	5.18 ± 0.20	13.10 ± 0.53b
S2	64.1 ± 1.9c	51.9 ± 1.0c	0.81 ± 0.01	5.36 ± 0.22	11.53 ± 0.46c
**System**	***	***	**	ns	***
**Salinity**	***	***	ns	ns	***
**System x Salinity**	ns	ns	ns	ns	ns

**Figure 2:**
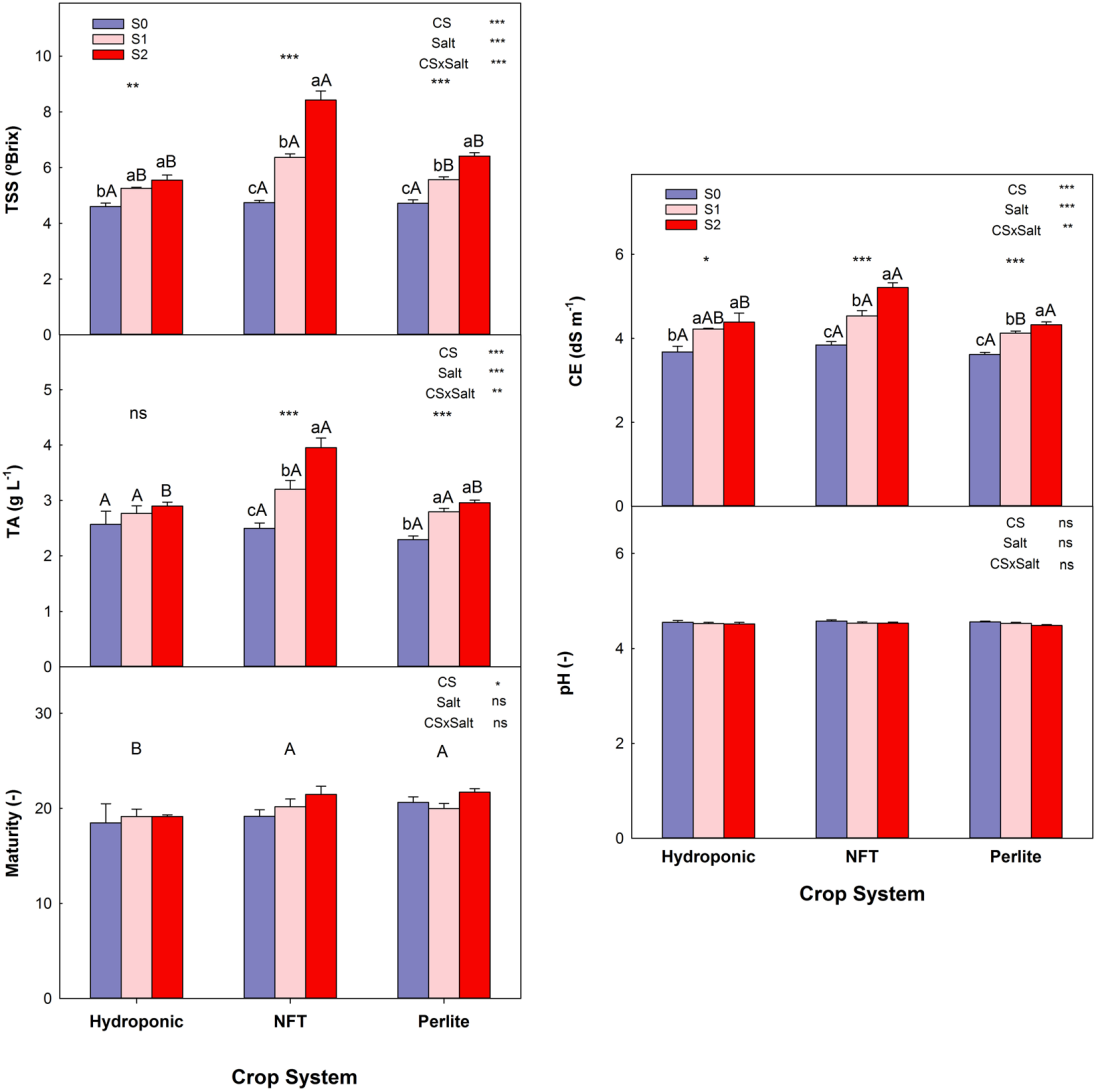
Chemical parameters of juice quality for tomato plants grown in three soilless culture systems and three salt treatments. The error bar indicates the standard error of the mean (n = 4–8). ***and “ns” indicate significant differences at p < 0.001 and non-significant differences, respectively. Values with different letters differ significantly at the 95% level, according to Tukey’s HSD test. Lowercase letters compare salinity treatments for each culture system. Uppercase letters compare culture systems for each salinity treatment.

Considering the parameters of juice quality - total soluble solids (TSS), titratable acidity (TA), EC, pH, and maturity - there were no differences among the fruits of the three systems when the S0 irrigation treatment was used (Fig. 2). Salinity showed a tendency to increase the TSS, TA, and EC in the juice of fruits of all three systems, but these increases were greatest in fruits from the NFT system. Thus, the highest values were obtained for salinity treatment S2: 8.4°Brix, 3.9 g·L^−1^, and 5.2 dS·m^−1^, respectively. The pH and the maturity index were not affected by salinity or by the cultivation system.

### Vegetative development of the plants

At the end of the experiment, regardless of the salinity treatment, the plants grown in the DFT system had the greatest vegetative growth, characterized by their high leaf and stem biomass and large total leaf area (Fig. 1B,D and F). Salinity decreased significantly the total biomass of the leaves and stem only in the plants cultivated in the NFT system. The decreases in these parameters were greater for the higher external salt concentration (S2), with reductions of 53 and 36% in leaf and stem biomass, respectively, relative to the control.

### Leaf concentrations of Cl^−^, Na^+^, and mineral nutrition

**Figure 3:**
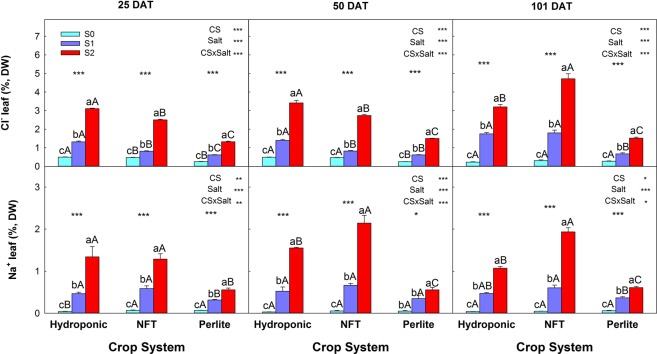
Leaf concentrations of Cl^−^ and Na^+^ of tomato plants grown in three soilless culture systems and three salt treatments. The error bar indicates the standard error of the mean (n = 6–9). *And ***indicate significant differences at p < 0.05 and p < 0.001, respectively. Values with different letters differ significantly at the 95% level, according to Tukey’s HSD test. Lowercase letters compare salinity treatments for each culture system. Uppercase letters compare culture systems for each salinity treatment.

In all cases, the concentrations of Cl^−^ and Na^+^ in the leaves increased progressively as the NaCl concentration in the NS increased. For all three samplings −25, 50, and 101 days after transplanting (DAT) - the lowest foliar concentrations of both Cl^−^ and Na^+^ were found in perlite-grown plants (Fig. 3). Regarding the other two systems, on the first two sampling dates the foliar concentration of Cl^−^ was higher in DFT-grown plants and at the third sampling it was superior in NFT-grown plants. In the case of Na^+^, for the S1 treatment there were no differences between the NFT and DFT, while for S2 the concentration of Na^+^ was higher for NFT-grown plants at 50 and 101 DAT.

**Figure 4:**
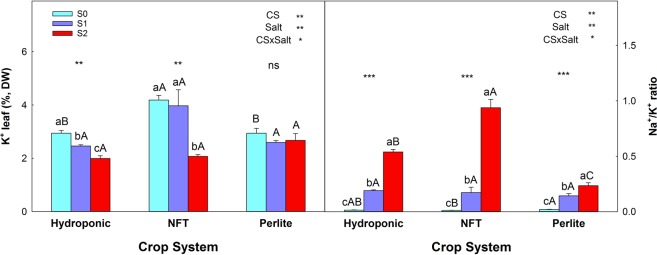
(**A**) Concentration of K^+^ and (**B**) the Na^+^/K^+^ ratio, at the end of the experiment, for leaves of tomato plants grown in three soilless culture systems and three salt treatments. The error bar indicates the standard error of the mean (n = 3–6). **And “ns” indicate significant differences at p < 0.01 and non-significant differences, respectively. Values with different letters differ significantly at the 95% level, according to Tukey’s HSD test. Lowercase letters compare salinity treatments for each culture system. Uppercase letters compare culture systems for each salinity treatment.

Under non-saline conditions, the foliar concentration of K^+^ was highest in plants grown with the NFT (Fig. 4A). Treatments S1 and S2 decreased the foliar concentration of K^+^ in the plants grown with DFT, and S2 reduced it in the plants of the NFT system. The Na^+^/K^+^ ratio increased with increasing NaCl concentration in the NS (Fig. 4B). In the S1 treatment there were no significant differences among the cultivation systems, but in S2 the values were highest for the NFT system, followed by DFT and perlite.

The concentration of all the major nutrients were maintained in the next ranges: Ca^2+^ (3.16–5.13% dry weight, DW), K^+^ (3.44–4.16% DW), P (0.74–1.36% DW), S (0.97–1.74% DW), and Mg^2+^ (0.37–0.53% DW) (Table 2).

**Table 2:** Values of the foliar concentrations of Ca^2+^, K^+^, P, S, and Mg^2+^ according to the soilless culture system and salinity (S0, S1, and S2). For each column, values not sharing a common letter differ significantly at the 95% level, according to Tukey’s HSD test. *, ***, and “ns” indicate significant differences at p < 0.05, p < 0.001, and non-significant differences, respectively, among salinity treatments for each soilless culture system. In all cases, the mean value ± the standard error is shown (n = 9–18).

	Ca^2+^ (%)	K^+^ (%)	P (%)	S (%)	Mg^2+^ (%)
**25 DDT**					
**Main factor: System**					
DFT	3.50 ± 0.12a	3.87 ± 0.14	0.59 ± 0.04b	0.97 ± 0.07b	0.48 ± 0.02b
NFT	3.05 ± 0.11b	3.98 ± 0.19	0.59 ± 0.04b	0.66 ± 0.03c	0.69 ± 0.01a
Perlite	3.02 ± 0.13b	4.05 ± 0.16	0.98 ± 0.09a	1.37 ± 0.05a	0.24 ± 0.04c
**Main factor: Salinity**					
S0	3.16 ± 0.14ab	4.16 ± 0.19	0.97 ± 0.08a	0.97 ± 0.05ab	0.53 ± 0.05a
S1	3.41 ± 0.13a	4.00 ± 0.10	0.68 ± 0.04b	1.10 ± 0.09a	0.47 ± 0.04a
S2	3.00 ± 0.11b	3.75 ± 0.18	0.50 ± 0.03c	0.92 ± 0.10b	0.40 ± 0.03b
**System**	*	ns	***	***	***
**Salinity**	ns	ns	***	*	***
**System x Salinity**	ns	***	***	**	*
**50 DDT**					
**Main factor: System**					
DFT	5.34 ± 0.19a	2.61 ± 0.31c	0.74 ± 0.06b	1.60 ± 0.09b	0.43 ± 0.02b
NFT	4.21 ± 0.27b	2.97 ± 0.46b	0.60 ± 0.06c	0.86 ± 0.09c	0.32 ± 0.02c
Perlite	5.04 ± 0.14a	3.43 ± 0.11a	0.98 ± 0.07a	1.91 ± 0.11a	0.66 ± 0.04a
**Main factor: Salinity**					
S0	4.97 ± 0.23	3.91 ± 0.22a	0.92 ± 0.07a	1.51 ± 0.11	0.51 ± 0.07
S1	4.79 ± 0.37	2.86 ± 0.17b	0.82 ± 0.07a	1.48 ± 0.20	0.44 ± 0.05
S2	4.82 ± 0.15	2.24 ± 0.33c	0.56 ± 0.06b	1.38 ± 0.22	0.47 ± 0.04
**System**	**	***	***	***	***
**Salinity**	ns	***	***	ns	ns
**System x Salinity**	ns	***	ns	*	ns
**101 DDT**					
**Main factor: System**					
DFT	5.83 ± 0.26a	2.47 ± 0.15b	0.88 ± 0.09b	1.61 ± 0.07b	0.37 ± 0.01b
NFT	4.58 ± 0.15c	3.49 ± 0.31a	0.94 ± 0.07b	0.98 ± 0.09c	0.24 ± 0.01c
Perlite	5.08 ± 0.11b	2.75 ± 0.11b	1.38 ± 0.06a	2.07 ± 0.07a	0.58 ± 0.03a
**Main factor: Salinity**					
S0	5.13 ± 0.24ab	3.44 ± 0.19a	1.36 ± 0.08a	1.74 ± 0.09a	0.46 ± 0.05a
S1	5.22 ± 0.16a	3.16 ± 0.32a	1.08 ± 0.07b	1.52 ± 0.17b	0.37 ± 0.03b
S2	4.76 ± 0.17b	2.31 ± 0.14b	0.84 ± 0.08c	1.37 ± 0.14b	0.37 ± 0.03b
**System**	***	**	***	***	***
**Salinity**	*	**	***	**	*
**System x Salinity**	ns	*	ns	*	ns

### Water relations and gas exchange parameters

**Figure 5:**
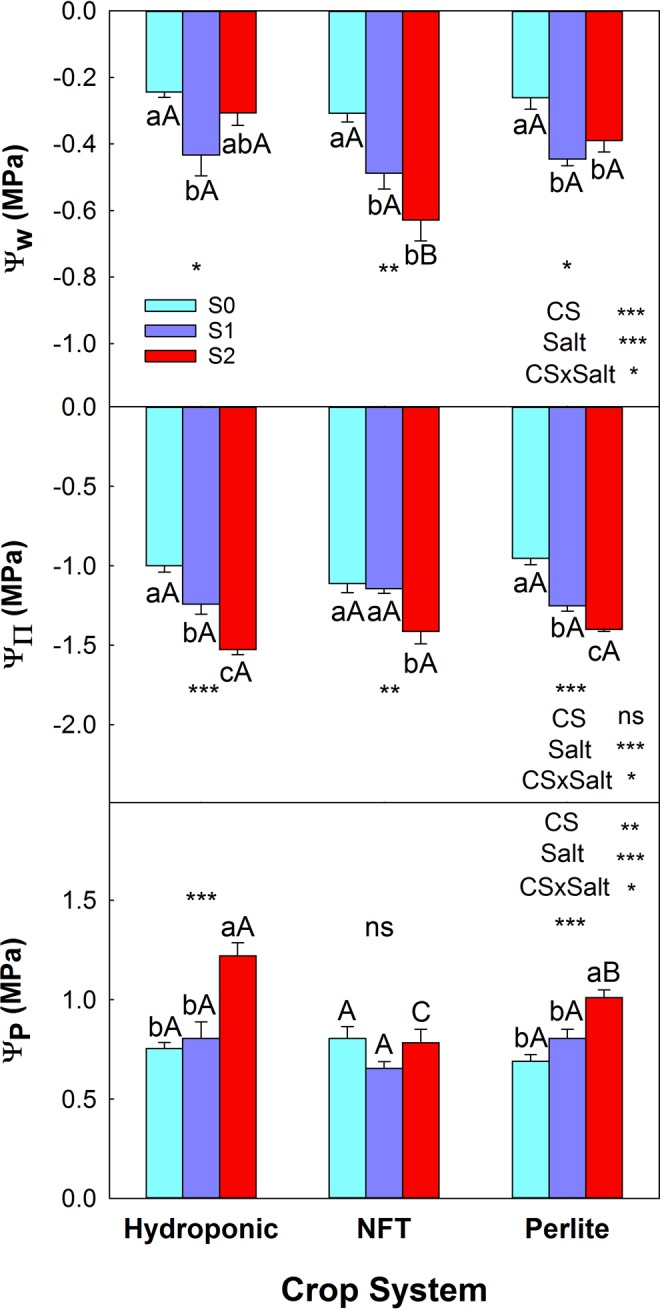
Parameters of leaf water relations of tomato plants grown in three soilless culture systems and three salt treatments. (**A**) Ψ_w_: water potential; (**B**) Ψ_π_: osmotic potential; (**C**) Ψ_P_: turgor potential. The error bar indicates the standard error of the mean (n = 3). *, **, ***, and “ns” indicate significant differences at p < 0.05, p < 0.01, p < 0.001, and non-significant differences, respectively. Values with different letters differ significantly at the 95% level, according to Tukey’s HSD test. Lowercase letters compare salinity treatments for each culture system. Uppercase letters compare culture systems for each salinity treatment.

The soilless culture system did not affect the values of the water relations (water potential, Ψ_w_, osmotic potential, Ψ_π_, and turgor potential, Ψ_P_) of the plants in the S0 treatment. Salinity decreased the value of Ψ_w_, due to a drop in Ψ_π_. The lowest value of Ψ_w_ was found in plants grown in the NFT system with treatment S2. In DFT and perlite, the S2 plants did not show a decline in Ψ_w_ and an increase in Ψ_P_ occurred (Fig. 5).

**Figure 6:**
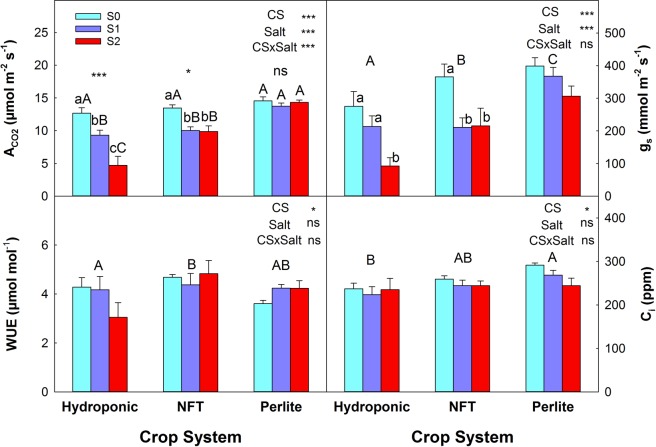
Leaf gas exchange parameters of tomato plants grown in three soilless culture systems and three salt treatments. (**A**) ACO_2_: net CO_2_ assimilation; (**B**) g_s_: stomatal conductance; (**C**) WUE_i_: instantaneous water use efficiency; (**D**) C_i_: substomatal concentration of CO_2_. The error bar indicates the standard error of the mean (n = 4–6). ***And “ns” indicate significant differences at p < 0.001 and non-significant differences, respectively. Values with different letters differ significantly at the 95% level, according to Tukey’s HSD test. Lowercase letters compare salinity treatments for each culture system. Uppercase letters compare culture systems for each salinity treatment.

As for the gas exchange parameters, under non-saline conditions the values of stomatal conductance (g_s_), substomatal CO_2_ concentration (C_i_), and net CO_2_ assimilation (ACO_2_) were highest for plants grown in perlite, followed by NFT and DFT. The instantaneous water use efficiency (WUE_i_) was higher for NFT-grown plants than in DFT and was lowest in perlite. The salinity treatments affected ACO_2_ and g_s_ (Fig. 6A and B). As external salinity increased, ACO_2_ and g_s_ decreased progressively in DFT-grown plants; in NFT-grown plants it also decreased with salinity but this decrease was similar in the two treatments, S1 and S2, while in perlite salinity did not affect ACO_2_. WUE_i_ and C_i_ were not affected by salinity (Fig. 6C and D).

## Discussion

From the data of total and marketable yield and leaf biomass obtained in this study, it can be concluded that, for tomato plants of the variety ‘Optima’ cultivated with non-saline NS, the DFT system is more productive. The values obtained are very similar to those found in other work with the same variety and soilless culture system^14^. The higher fruits yield derived from a greater mean fresh weight per fruit, while the number of fruits was not affected by the soilless culture system. The results for the fruit quality parameters were very similar in the three soilless culture systems. This indicates that the fruits on plants of the variety ‘Optima’ showed low sensitivity to the different environmental conditions caused by the soilless culture systems. In addition, regarding mineral nutrition, all the major nutrients were maintained in a normal range for tomato plants grown in non-saline conditions^21^ (Table 2). However, leaf Ca^2+^ and P concentrations were higher than normal (P: 0.20–0.60% DW, Ca^2+^: 1.00–2.00% DW), suggesting that “Optima” plants could be a variety that accumulates Ca^2+^ and P. The ACO_2_ and Ψ_w_ were also similar for the plants of the three systems, with overall mean values of 12 mmol·m^−2^·s^−1^ and −0.2 MPa, respectively. Taking into account that the management of the fertigation was optimum in each culture system to reach the maximum potential of the crop, all these data suggest that the higher yield in DFT was due to the fact that the plants had available water and nutrients at all times. In the case of perlite and NFT, although irrigated on demand according to plant size, during the inter-irrigation periods the plants could have undergone osmotic and/or hydric stress, with negative effects on the total yield and the vegetative development of the crop. In most experiments comparing different substrates for horticultural crops, the differences were not so marked as in our experiment^22,23^. This could be because in our assay we compared very different systems: in DFT the roots are always submerged in the NS and therefore the water and nutrients are easily available; in NFT, although it is a hydroponic-like system, the roots are only in contact with the NS when the irrigation is applied, the rest of the time they remain naked; and perlite is a solid, inert substrate in which both the water content and the salt concentration can vary widely between irrigation events. Moreover, the medium in which the roots are located differs greatly among these systems; hence, the morphological and physiological properties of the roots can also differ, determining the yield response.

When choosing a soilless culture system, another important aspect is the agricultural efficiency of water use (WUE_agri_, kg·m^−3^, determined as the relationship between yield and the volume of NS used for irrigation). In our experiment, the total amount of NS used in the control treatment was 181, 176, and 195 L·plant^−1^, for the DFT, NFT, and perlite, respectively. Thus, to obtain 1 kg of fruit, 36 L (DFT), 46 L (NFT), or 46 L (perlite) of NS were required.

The marketable yield of plants of the tomato variety ‘Optima’ decreased progressively as the EC of the NS increased, and this reduction was similar in the three soilless culture systems tested - being, on average, 35% and 58% at 40 mM (6.0 dS·m^−1^) and 80 mM NaCl (10.0 dS·m^−1^), respectively, relative to the control treatment (S0). This means that, with the irrigation management used in this work, the soilless culture system hardly affected the behavior of the plants under saline conditions, plants cultivated with DFT being the most productive under these conditions also. The reduction of the marketable yield by salinity was due to a fall in the average fruit weight, which declined from 188 g for the control treatment to 142 and 123 g for treatments S1 and S2, respectively. However, the number of fruits per plant was only reduced by salinity in tomato plants cultivated with the NFT, being 23, 17, and 9 for treatments S0, S1, and S2, respectively. In general, the reduction in the yield of tomato plants when irrigated with water of moderate salinity is due mainly to a decrease in the average weight of the fruits, whereas at high salinity it arises from decreases in both the average fruit weight and the number of fruits^24–27^. This indicates that plants in the NFT system, in the period between two consecutive irrigations, could have suffered a more intense osmotic effect than those in the other two systems - leading to a loss of fruits, due to either poor flowering or the drop of recently-set fruits^28^.

The data from this experiment show that, for the tomato variety ‘Optima’, a yield loss of approximately 7.3% occurred for each increase of 1.0 dS·m^−1^ in the EC of the NS above 2.0 dS·m^−1^. This indicates that this variety can be considered moderately sensitive to salinity, like ‘Clarance’ or cherry varieties^27,29^ - which are more sensitive than the hybrid varieties ‘HC01’^30^ and ‘Durinta’^31^.

Salinity, in addition to affecting fruit size, also affected certain organoleptic characteristics such as firmness, TSS, and TA. The decrease in firmness occurred in the fruits from all three soilless culture systems, and could be due to the decrease in the Ca^2+^ concentration in the fruits (data not shown). Normally, the Ca^2+^ concentration is decreased by external salinity because this element moves through the xylem following the transpiration stream, which is reduced by the osmotic effect of salinity in the root zone. The values of the fruit juice quality parameters were improved by salinity; specifically, increases in TSS and TA were observed, the highest values of both being found in the fruits of plants cultivated in the NFT system with the S2 treatment. The increases in the levels of organic solutes - sugars, organic acids, etc. - under saline conditions are due to the fact that plants adjust osmotically to avoid the harmful osmotic effect of salinity, and thus avoid the dehydration of different tissues^32^. The fact that TSS and TA increased with salinity much more in the fruits of plants grown in the NFT system could be related mainly to two factors: i) the fruits of these plants were smaller, so a concentration effect could have been produced for the sugars, and ii) the osmotic effect of salinity combined with the lack of water between the irrigation events could have caused a greater increase in the synthesis of organic solutes in these plants.

One of the negative effects of salinity on tomato plants is the toxic effect of Cl^−^ and Na^+^ on leaves^33,34^. In this experiment, increasing the concentration of NaCl in the NS increased progressively the concentrations of these ions in the leaves. So, the high leaf concentrations of these ions contributed to the diminished yield of the plants. However, at the end of the experiment, differences among the systems were observed. Thus, the plants grown in the NFT system had higher concentrations of Cl^−^ and Na^+^ (4.71 and 1.94% DW, respectively) than the plants grown with DFT (3.20 and 1.07% DW, respectively) or in perlite (1.52 and 0.61% DW, respectively). However, in spite of these differences, the effects of salinity on the marketable yield were similar in all three systems, indicating that the loss of yield in each system was due to the combined action of the toxic and osmotic effects of salinity. A significant negative effect of Cl^−^ and/or Na^+^ toxicity on vegetative development was observed only in NFT plants, the ones that exhibited the highest concentrations of these ions. In the NFT system, in the period between two consecutive irrigations, the water taken up by the roots and the evapotranspiration of part of the water could have increased the Na^+^ and Cl^−^ concentration in the NS and hence the root uptake of these ions.

The presence of elevated levels of Cl^−^ and/or Na^+^ in the solution around the roots influences the uptake of nutrients by the plant through interactions, due to competition between nutrients, or by altering the permeability of membranes^35^. In our experiment, salinity increased the Na^+^/K^+^ ratio in the plants of the three soilless culture systems, but in the cases of DFT and NFT this was due to both an increase in tissue Na^+^ and a decrease in the K^+^ concentration. In treatment S2, this ratio was 0.22 for plants in perlite, 0.53 in DFT, and 0.93 for NFT (control treatment, Na^+^/K^+^  = 0.014). The reduction of root K^+^ uptake by Na^+^ is a competitive process. Although plants have a high selectivity for K^+^ over Na^+^, excessive amounts of Na^+^ may have a negative effect on plants^36^ and this could have been more pronounced in NFT plants. So, the decrease in the K^+^ concentration could be related with the reduction of the number of fruits per plant^37^. Potassium and Fe are the elements that are of greatest importance in soilless culture systems in terms of tomato yield and fruit quality. Screening to determine the best doses of both elements can improve marketable yield and fruit quality^38^.

Another negative effect caused by the excess of NaCl in the nutrient solution was the decrease of the foliar concentration of P, and this was found to be the case in all three soilless systems on the three sampling dates (Table 2). The general tendency was that the increase in salinity of the nutrient solution produced a decrease in the concentration of P in the leaves. It is well known that the salinity in crops induces a decrease in the uptake of essential nutrients such as phosphorus and potassium^39^. In the plants of the variety Optima, as they already have a high concentration of P *per se*, this decrease did not overcome the levels of deficiency (<0.2% DW)^21^, although any nutritional disequilibrium of P with respect to the other nutrients could have caused a negative effect in the production and vegetative development of the plants^39^. It should also be taken into account that the high concentration of P in the leaves could have decreased the functioning of Zn, and although this nutrient was found in normal concentrations (20 ppm, data not shown), this could have caused an additional stress factor with a negative impact on abiotic stress tolerance^40^.

The ACO_2_ in tomato plants can be affected adversely by salinity^41^. In our experiment ACO_2_ was decreased by salinity in plants grown with DFT or in the NFT system, but not in perlite. In addition, a significant, linear correlation of ACO_2_ was observed with the leaf Cl^−^ concentration and with g_s_; however, ACO_2_ was not correlated with the Na^+^ concentration or C_i_ (data not shown). This response is not novel as it has been reported for other crops^42^. In addition, our results suggest that the reduction of ACO_2_ by salinity was due to alterations in the metabolic processes of photosynthesis and not to stomatal factors. Although ACO_2_ and g_s_ were reduced by salinity, the fact that the C_i_ was similar to that of control plants indicates that ACO_2_ was not limited by a low concentration of CO_2_ in the mesophyll cells caused by stomatal closure^43^. This type of ACO_2_ response in relation to stomatal and non-stomatal factors in tomato plants seems to depend on the variety. It has been observed that metabolic factors are more important than stomatal ones in the tomato varieties ‘RAF’ and ‘Leader’, but in ‘Daniela’ stomatal factors are responsible for the reduction of ACO_2_^44^. In addition, comparing the ACO_2_ data with the Cl^−^ and Na^+^ concentrations, the reduction of ACO_2_ appeared to be related to Cl^−^ rather than Na^+^ toxicity, as a negative and significant correlation was obtained between ACO_2_ and Cl^−^, but not Na^+^. Also, the plants grown in perlite had the lowest concentration of Cl^−^ and their ACO_2_ was not affected by salinity.

One of the detrimental effects of salinity on plants is the osmotic effect of salt accumulation, both in the soil solution and inside the cells^45^. The water relations data obtained in this experiment indicate that the plants grown in the NFT system were those whose water status was affected most: under saline conditions they had the lowest values of Ψ_w_ and Ψ_P_. In general, however, the osmotic effect of salinity did not cause leaf dehydration, as indicated by the leaf relative water content (data not shown) and Ψ_P_. These plants could have prevented leaf dehydration through stomatal closure and osmotic adjustment, in which the accumulation of organic ions and solutes allows plants to reduce their Ψ_w_.

In conclusion, the marketable yield was affected by the soilless culture system and decreased for increasing salinity in the nutrient solution. So, regarding soilless culture system fruit production (kg·plant^−1^) followed in the order: DFT > perlite > NFT, and this was similar for each salinity treatments. Regarding the fruits, the salinity treatments improved their quality by increasing the total soluble solids and titratable acidity, particularly in the NFT system. Salinity caused changes in the water status of the plants, toxicity due to Cl^−^ and Na^+^, and nutritional imbalances that altered the growth and the physiology of the plants; these effects were influenced by the soilless culture system. Although plants under NFT, had less production, this technique could have some advantage such as avoiding the contamination of underground soils and aquifers due to the release of fertilizers. However, it is necessary to continue studying the agronomic behavior of the tomato produced with this technique using irrigation water of different quality, in order to determine its effect on water and nutrient savings.

## Methods

### Plant material and growth conditions

The experiment was carried out between April and July 2014 with ‘Optima’ tomato plants (*Solanum lycopersicum* L.) grown in a greenhouse located at the experimental station of the CEBAS (Santomera, Murcia, Spain; 38°6′26″N, 1°2′7″W). The greenhouse was the multitunnel type, with polycarbonate sides and a polyethylene roof. The covered area of the greenhouse was 650 m^2^ and the height up to the channel was 4.5 m. For control of the internal climate, the greenhouse had an indirect-combustion hot air generator (SIAL Mirage 65, Munters, Chiusavecchia, Genoa, Italy), fans, side and overhead ventilation, a cooling system (Novedades Agrícolas, Murcia, Spain), a shade mesh (Aluminet 30%, Novedades Agrícolas, Murcia, Spain), and a humidification unit with an air compressor (Ingersoll Rand SSR, Dublin, Ireland). The values of radiation, temperature, and relative humidity were recorded periodically by sensors located at a height of 1.5 m inside the greenhouse. The mean values over the course of the experiment were 381 W·m^−2^, 24 °C, and 66.3%, respectively (Table 3).

**Table 3:** Climate and irrigation in function of the soilless system and the saline treatment. Med: mean; Min: minimum; Max: maximum. The total consumption of water per plant and the maximum electrical conductivity (EC) reached by the nutrient solution (NS) in the root zone. S0 (EC = 2.2 dS·m^−1^), S1 (EC = 6.3 dS·m^−1^), and S2 (EC = 10.2 dS·m^−1^) are salt treatments with initial different values of EC in the NS.

	Temperature (°C)			Relativity humidity (%)			Radiation (W·m^−2^)		
	Med	Min	Max	Med	Min	Max	Med	Min	Max
April	20.3	12.2	31.9	67.4	53.4	91.6	320	148	399
May	22.3	16.4	32.1	68.1	59.1	89.6	361	130	442
June	26.1	20.2	28.1	64.5	52.6	92.1	412	129	532
July	27.1	22.3	29.2	65.2	52.1	94.5	433	136	584
	**Water consumption per plant (L)**			**Root medium EC (dS·m** ^− **1**^ **)**					
	**DFT**	**NFT**	**Perlite**	**DFT**	**NFT**	**Perlite**			
**S0**	181	176	195	2.1	2.3	3.3			
**S1**	165	168	172	6.6	6.3	10.5			
**S2**	132	164	152	10.3	10.2	15.0			

The plants, from a commercial nursery (Baby Plant, SL, Santomera, Murcia, Spain), were transplanted (14 days after sowing) into the three soilless culture systems selected for this experiment: DFT, NFT, and perlite. The NS was the same for all the plants, consisting of 6 mM KNO_3_, 4 mM Ca(NO_3_)_2_, 1 mM KH_2_PO_4_, 1 mM MgSO_4_, 20 μM Fe^3+^ masquolate, 25 μM H_3_BO_3_, 2 μM MnSO_4_, 2 μM ZnSO_4_, 0.5 μM CuSO_4_, and 0.5 μM (NH_4_)_6_Mo_7_O_24_·4H_2_O, with pH = 6.0. At 14 DAT, the plants of each culture system were divided into three groups, to which distinct salinity treatments were applied: control (S0), 40 mM NaCl (S1), and 80 mM NaCl (S2). To avoid possible osmotic shock, the initial NaCl concentration in treatments S1 and S2 was 20 mM; it was increased daily by 20 mM to the desired final concentration.

**Figure 7:**
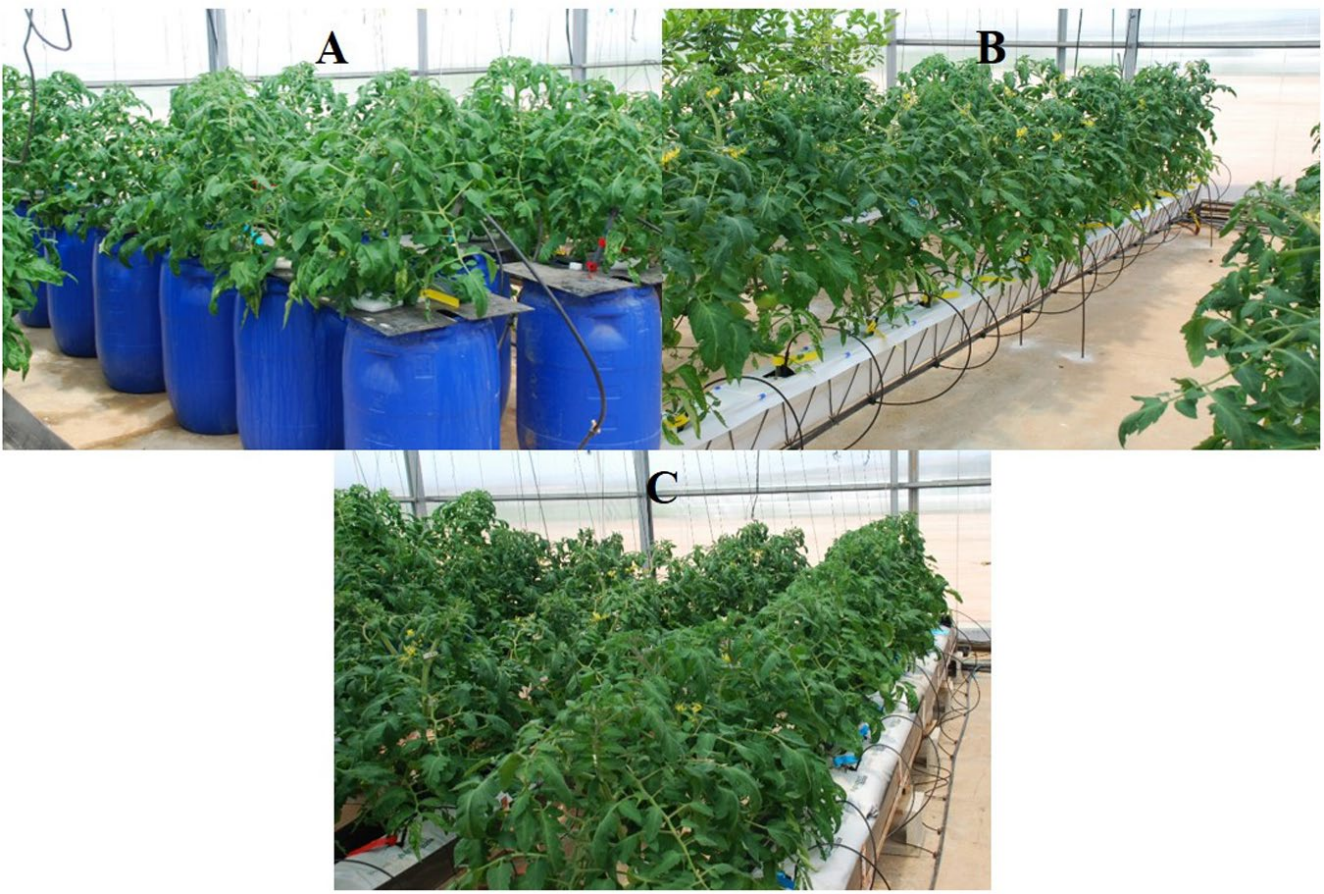
The three soilless culture systems in which the plants were grown. Deep flow technique (**A**), Nutrient film technique (**C**), and Perlite (**C**).

The DFT, with continuous aeration, was performed in 24 polyvinyl containers of 120-L capacity. They were sealed with a black plastic plate that held the plant (Fig. 7A). The volume of the NS was checked three times a week and kept constant by adding deionized water; the NS nutrients and pH were analyzed weekly so that their baseline levels could be restored by adding fertilizers and NaOH, respectively. The NFT was performed with six V-shaped multi-bands (the New Growing System, NGS©), planting 12 plants per line (Fig. 7B). In the perlite system, 24 bags, each with a capacity of 40 L (1.20 × 0.22 × 0.15 m), were used, with three plants in each (Fig. 7C). Irrigation was performed - without recycling - according to the management practices recommended for greenhouse crops grown in perlite in Almeria^46^, using self-compensating and self-draining drippers set at 3.0 L·h^−1^, with weekly programming, taking into account the volume drained and its EC. For each irrigation event the drainage solution represented an initial 15% of the total volume applied, and when the EC of the drainage solution was progressively increased the irrigation volume was increased by 5% for every 1.0 dS·m^−1^ increment in EC, up to a maximum of 30% - to avoid excessive consumption of water. The drainage solution was stored in a 1000-L tank for its recirculation in a closed system. Irrigation was performed with self-compensating and self-draining drippers set at 8.0 L·h^−1^. The EC of the solution in the tank was controlled daily by adding deionized water, to maintain the EC at 2.2, 6.3, and 10.2 dS·m^−1^ for treatments S0, S1, and S2, respectively. Water with ECs of 6.3 dS·m^−1^ and 10.2 dS·m^−1^ are used by the tomato growers in the Spanish southeast, and are considered to have moderate and severe salt content, respectively. The nutrients and pH were also checked weekly and restored when necessary. During the first 30 DAT, in each 20-minute period the plants were irrigated for 5 minutes, but not during the following 15 minutes. Subsequently, the interval without irrigation was shortened to 10 minutes and finally to 5 minutes.

The duration of the experiment was 108 days. The cultivation techniques used were similar to those of commercial greenhouses in the area. The experimental design was bifactorial (3 soilless culture systems × 3 salinity treatments; Table 4) in two identical blocks, in which the treatments were distributed randomly.

**Table 4:** Soilless culture systems (deep flow technique, DFT, nutrient film technique, NFT, and perlite), and concentration of NaCl and electrical conductivity (EC) of nutrient solutions used in the assayed. The experimental design was bifactorial with 3 soilless culture systems × 3 salt treatments.

Soilless system	Salt Treatment	NaCl (mM)	EC (dS m^−1^)
DFT	S0	0	2.2
	S1	40	6.3
	S2	80	10.2
NFT	S0	0	2.2
	S1	40	6.3
	S2	80	10.2
Perlite	S0	0	2.2
	S1	40	6.3
	S2	80	10.2

### Yield parameters and fruit quality

The total fruit yield per plant (kg and number of fruits) was determined by harvesting the fruits daily during the experiment, when they reached their characteristic red color, determining fresh weight and caliber individually for each plant. According to these values, the fruits were classified as marketable or non-marketable (fresh weight < 70 g, with apical rotting, cracking, or some type of deformation or mechanical damage).

The quality parameters were measured in 24 fruits for each combination of soilless culture system and salinity treatment, considering an experimental unit as three fruits harvested per plant (n = 8). The fruits analyzed were from clusters two and six. To evaluate the quality of the fruit, the shape index (the ratio of the equatorial to the longitudinal diameter), color index (Minolta CR300, Tokyo, Japan), hardness (Bertuzzi FT-327, Facchini, Alfonsine, Italy), and firmness (TA XT Plus, TTC, Hamilton, Massachusetts) were determined. In the fruit juice, the pH, EC, total soluble solids (TSS, Atago N1, Minato-ku, Tokyo, Japan), and titratable acidity (TA, potentiometric titration to pH 8.1; AOAC, 1984) were measured.

### Vegetative growth and mineral composition of the plant tissues

To determine the nutritional status of the plants, leaf samples (5 mature leaves per plant, 8 plants per treatment) were taken 25, 50, and 101 DAT. The leaves were washed with deionized water, dried at 65 °C for 48 hours, ground, and analyzed by inductively coupled plasma spectrometry (Iris Intrepid II, Thermo Electron Corporation, Franklin, Massachusetts, USA) after digestion in a CERM microwave (Mars Xpress, North Carolina, USA). At the end of the experiment, after harvesting the last fruits, eight plants per treatment were harvested and the fresh weight and leaf area (LICOR-3100C, Lincoln, Nebraska, USA) of each plant were measured. In addition, these plants were oven-dried to determine the DW of each of the plant components (leaves, stem, and roots).

### Parameters of water relations and gas exchange

At 90 DAT, a physiological study - of leaf water relations, leaf gas exchange, and chlorophyll fluorescence parameters - was carried out. The measurements of water relations were performed using a single mature leaf from the mid-stem region of each of six replicate plants. The pre-dawn leaf water potential (Ψ_w_) was measured using a Scholander-type pressure chamber (PMS Instruments, Corvallis, Oregon, USA^47^). After the measurement of Ψ_w_, the leaves were immediately wrapped tightly in aluminum foil, frozen by immersion in liquid nitrogen, and subsequently stored in airtight plastic bags at −18 °C. After thawing, the osmotic potential (Ψ_π_) of the extracted sap was measured at 25 ± 1 °C with an osmometer (Digital Osmometer, Wescor, Logan, Utah, USA). The turgor potential (Ψ_P_) was calculated as the difference between Ψ_w_ and Ψ_π_.

The net assimilation of CO_2_ (ACO_2_), stomatal conductance (g_s_), leaf transpiration (E_leaf_), and ACO_2_/E_leaf_ ratio were measured on the same day as the leaf water relations, using a portable photosynthesis system (CIRAS-2, PP-System, Amesbury, Massachusetts, USA). During all of these measurements, the CO_2_ concentration was 380 ± 5 ppm, the PAR was 1000 µmol·m^−2^·s^−1^, and the leaf temperature and leaf-to-air vapor pressure deficit were 29 °C and 1.9 kPa, respectively. All measurements were performed in the morning, from 09:00 to 12:00 h, using a single mature leaf in the mid-stem region of each of the six replicate plants per treatment.

### Statistical analysis

With the data of the experiment a trifactorial analysis of variance (ANOVA) (salinity x soilless culture system x block) was performed. Because the block effect was not significant (p > 0.05), only the results of a bifactorial ANOVA, with soilless culture system and salt treatment as the main factors, are reported (SPSS statistical package, Chicago, Illinois, USA). When this interaction was significant (p < 0.05), the treatment means were separated by Tukey’s HSD multiple-range test, using lowercase letters to indicate significant differences between salt treatments for each soilless culture system, and uppercase letters to indicate significant differences between soilless culture systems for each salt treatment.

All this material and methods have been described in^48^.
